# Novel Anti-fibrotic Therapies

**DOI:** 10.3389/fphar.2017.00318

**Published:** 2017-05-31

**Authors:** Benita L. McVicker, Robert G. Bennett

**Affiliations:** ^1^Research Service, VA Nebraska-Western Iowa Health Care System, OmahaNE, United States; ^2^Division of Gastroenterology and Hepatology, University of Nebraska Medical Center, OmahaNE, United States; ^3^The Division of Diabetes, Endocrinology, and Metabolism, Department of Internal Medicine, University of Nebraska Medical Center, OmahaNE, United States; ^4^Department of Biochemistry and Molecular Biology, University of Nebraska Medical Center, OmahaNE, United States

**Keywords:** fibrosis, cardiovascular disease, antifibrotic agents, bone morphogenic protein-7, micro-RNA, relaxin, peroxisome proliferator-activated receptors

## Abstract

Fibrosis is a major player in cardiovascular disease, both as a contributor to the development of disease, as well as a post-injury response that drives progression. Despite the identification of many mechanisms responsible for cardiovascular fibrosis, to date no treatments have emerged that have effectively reduced the excess deposition of extracellular matrix associated with fibrotic conditions. Novel treatments have recently been identified that hold promise as potential therapeutic agents for cardiovascular diseases associated with fibrosis, as well as other fibrotic conditions. The purpose of this review is to provide an overview of emerging antifibrotic agents that have shown encouraging results in preclinical or early clinical studies, but have not yet been approved for use in human disease. One of these agents is bone morphogenetic protein-7 (BMP7), which has beneficial effects in multiple models of fibrotic disease. Another approach discussed involves altering the levels of micro-RNA (miR) species, including miR-29 and miR-101, which regulate the expression of fibrosis-related gene targets. Further, the antifibrotic potential of agonists of the peroxisome proliferator-activated receptors will be discussed. Finally, evidence will be reviewed in support of the polypeptide hormone relaxin. Relaxin is long known for its extracellular remodeling properties in pregnancy, and is rapidly emerging as an effective antifibrotic agent in a number of organ systems. Moreover, relaxin has potent vascular and renal effects that make it a particularly attractive approach for the treatment of cardiovascular diseases. In each case, the mechanism of action and the applicability to various fibrotic diseases will be discussed.

## Introduction

Fibrosis is a critical stage of many chronic diseases that can lead to organ dysfunction, illness and death. The burden associated with fibrosis is staggering with nearly half of all deaths in the United States attributed to fibrotic diseases including liver, lung, kidney, and heart disorders (Wynn, 2008; Rosenbloom et al., 2010). Unfortunately, the impact of fibrosis on mortality and morbidity rates has not been countered by effective treatments. Decades of research have identified many potential targets to combat fibrotic tissue damage, yet there currently are no effective therapies or FDA approved antifibrotic agents. This review highlights the potential of four agents that have emerged from basic science testing that show promise as translatable options for the treatment of fibrotic diseases in humans.

The development of antifibrotic therapies relies on the comprehensive understanding of profibrogenic mechanisms in multiple organ systems as well as disease-specific locations. The global and foremost mechanism involved in fibrosis is the activation of myofibroblasts resulting in the excessive and often continual production of extracellular matrix (ECM) components, the foundation of scar formation (McAnulty, 2007; Herzog and Bucala, 2010). Myofibroblast activation is initiated in chronic inflammatory diseases where healthy wound healing in response to injury is not controlled, resolved, or is repetitively stimulated by an inciting factor. As a result, differentiated fibroblasts are stimulated to be in a proliferative, activated state producing ECM components. In the last 25 years, heightened research has identified potential targetable pathways and related individual factors that are involved in the differentiation of quiescent fibroblasts and the persistent activation of myofibroblasts during fibrogenesis. These include an array of cytokines (e.g., interleukins, IL-1, IL-6, IL-25, IL-33; and tumor necrosis factor-α, TNF-α) that are produced as a consequence of inflammation and epithelial and endothelial tissue damage (Rosenbloom et al., 2013). The burst of inflammatory mediators can directly stimulate fibroblast activation as well as influence downstream adaptive and innate immune mechanisms (Wynn and Ramalingam, 2012). For example, a stimulated adaptive immune system can affect a complex array of T and B cell activities resulting in the resolution as well as promotion of fibrosis via interferon gamma (IFN-γ) signaling (Gurujeyalakshmi and Giri, 1995). Innate immune activation is similarly complex involving responses of multiple cell types (e.g., macrophages, neutrophils and mast cells) along with their associated cytokines and growth factors that drive a fibrotic signaling cascade (Barrientos et al., 2008; Lech and Anders, 2013). Among the factors found to be associated with innate immune cells, TNF-α and IL-1β are noted profibrotic mediators along with transforming growth factor-beta (TGF-β), which has long been identified as a key factor in fibrosis (Roberts et al., 1988). Altogether, inflammatory and immune cell factors ultimately stimulate myofibroblasts to produce smooth muscle actin and matrix components such as collagen and fibronectin. The goal to inhibit profibrogenic factors or pathways has been the focus of research for years. Unfortunately, successful translation of effective antifibrotic treatments to humans has been limited due to inefficient and off-target effects. However, recent preclinical assessments of several agents show promise in achieving an attainable inhibition of fibroblast activation and ECM expression through mechanisms involving the blockade of signaling receptors, TGF-β stimulation, or translation of fibrogenic genes (Friedman et al., 2013; Gourdie et al., 2016).

Of the various mechanisms that have been studied to inhibit fibroproliferative events, strategies to target the activation of the myofibroblasts are emerging with high potential. Recent advances have been noted in the understanding and importance of particular factors in the inhibition and/or resolution of fibrosis. Specifically, excitement has been noted for the antifibrotic potential of bone morphogenetic protein-7 (BMP7); micro-RNAs; peroxisome proliferator-activated receptor (PPAR) signaling pathways; and the hormone relaxin. A strong indication for the use of these factors has been shown in studies of atrial fibrosis, heart failure, renal disease and cirrhosis of the liver. Acknowledging the importance and emergent nature of cardiovascular disease, this review will discuss the antifibrotic potential of the above agents in a disease-specific manner with emphasis on cardiac injury, as well their applicability as a broad intervention.

## The Antifibrotic Therapeutic Role of BMP7

Bone morphogenetic protein-7 was discovered nearly 30 years ago as a critical factor in development and bone formation (Ozkaynak et al., 1990). Interestingly, BMP7 was also determined to be part of the TGF-β family. Considering the association of BMP7 with the fibrogenic factor TGF-β, the intervening years since its identification has produced a wealth of information related to mechanisms and the targetability of BMP7 pathways in fibrotic disease. To date, basic science discoveries have detailed the importance of BMP7 in organ homeostasis and specifically as an opposing mechanism to the profibrogenic actions of TGF-β. However, the translation of BMP7 as an antifibrotic agent remains in development. The following overview highlights the functional significance of BMP7 and related signaling mechanisms that may lead to translational successes and clinically relevant treatments for fibrosis.

BMP7 is structurally and functionally similar to members of the TGF-β superfamily (Weiskirchen et al., 2009). Structurally, BMP7 has a related modular form and sequence to TGF family members including a C-terminal biologically active region that is highly homologous to TGF-β. Also, similar to other family members, BMP7 engages with serine/threonine kinase receptors leading to the initiation of signal transduction cascades (Massague et al., 2005). Details of BMP7-mediated signaling have been eloquently characterized describing a variety of gene responses that are induced as a result of BMP7 activity (Weiskirchen et al., 2009). Outcomes of BMP7 signaling were shown to include the regulation of genes associated with embryonic development including kidney, eye and skeleton formation. Moreover, BMP7 signaling significantly influences organ homeostasis and importantly, the regulation of antifibrotic mechanisms. A summary of the major and most recent reports on the effectiveness of BMP7 treatment is presented in **Table 1** and is discussed below.

**Table 1:** Antifibrotic therapeutic potential of targeting BMP7 signaling.

Fibrotic disease	Treatment	Findings	Study
Cardiac	rhBMP7	Inhibition of EMT	Zeisberg E.M. et al., 2007
	rhBMP7	Suppression of left ventricular remodeling	Merino et al., 2016
Renal	BMP7	Restoration of BMP7 levels; partial reversal of diabetic-induced kidney disease	Wang et al., 2003
	THR-123	Induction of BMP receptor activin-like kinase 3 signaling; suppression of inflammation, EMT	Sugimoto et al., 2012
Hepatic	AVV-BMP7	Suppression of carbon tetrachloride-induced fibrosis and promotion of hepatocyte regeneration	Hao et al., 2012
	rhBMP7	Reduction of hepato-schistosomiasis-associated fibrosis via antagonism of TGF-β signaling	Chen et al., 2013
	Cpd 861	Upregulation and activation of BMP7 signaling	Hou et al., 2016
Pulmonary	Tilorone	Enhancement of BMP7 expression and signaling in lung epithelial cells	Lepparanta et al., 2013
	rhBMP7	Reversal of TGF-β-mediated myofibroblast differentiation regulated by hyaluronan	Midgley et al., 2015
	BMP7	Attenuation of silica-induced fibrosis via regulation of BMP signaling	Liang et al., 2016
Corneal	ITF2357	Activation of Id3 and BMP7 levels	Lim et al., 2016

*BMP7, Bone Morphogenetic Protein 7; rhBMP7, recombinant human BMP7; EMT, endothelial-mesenchymal transition; THR-123, small peptide BMP agonist, Thrasos Therapeutics, Canada; AVV-BMP7, adeno-associated virus carrying BMP7; Cpd 861, herbal compound 861; Tilorone, Tilorone dihydrochloride (CAS 27591-69-1); TGF-β, transforming growth factor beta; ITF2357, (diethyl-[6-(4-hydroxycarbamoyl-phenyl carbamoyloxymethyl)-naphthalen-2-yl methyl]-ammonium chloride (Givinostat).*

In early studies using BMP7 deficient mice it was shown that a key factor in the antifibrotic effect of BMP7 involved the control of epithelial-to-mesenchymal transition (EMT) and TGF-β profibrogenic signaling in multiple organs (kidney, heart, liver, lung, and eye) (Luo et al., 1995; Zeisberg E.M. et al., 2007; Zeisberg M. et al., 2007; Myllarniemi et al., 2008). It is well known that TGF-β is a central inducer of myofibroblast activation and that TGF-β-mediated EMT is important in the transformation of fibroblasts involved in wound healing responses and fibrosis. Results from multiple fibrotic disease models demonstrated that BMP7 expression is downregulated during disease and that the restoration of BMP7 expression or treatment with recombinant protein resulted in the prevention or alleviation of fibrosis (Weiskirchen et al., 2009). The protective role of BMP7 was found to correlate with the inhibition of TGF-β-mediated profibrotic signaling. Despite the promising reports from various animal studies, the beneficial effects of exogenous BMP7 were found to be variable. This was evident from studies which failed to demonstrate an antifibrotic benefit of BMP7 as well as the indication that BMP7 expression in fact correlated with fibrotic disease (Ikeda et al., 2004; Tacke et al., 2007). However, the role of BMP7 as an opposing mechanism to the profibrogenic effects of TGF-β signaling remains clinically relevant, especially with the development of alternative strategies to alter BMP signaling through the use of small molecule inhibitors or agonists. Therefore, efforts continue to define the therapeutic effectiveness of BMP7 through more in-depth investigations including *in vivo* analyses, comparative studies and preliminary efficacy trials (Sugimoto et al., 2012; Lepparanta et al., 2013; Midgley et al., 2015; Lim et al., 2016).

**FIGURE 1:**
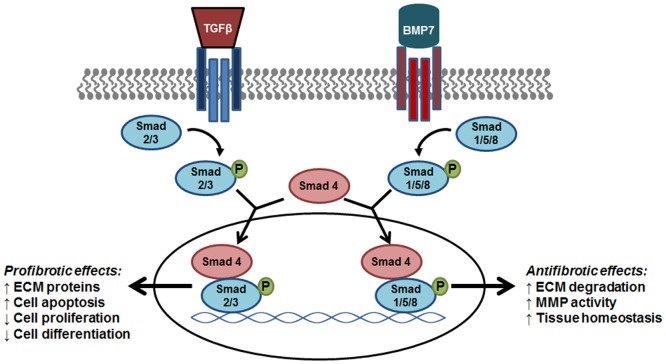
The antifibrotic effect of BMP7/Smad signaling. As members of the same family, BMP7 and TGF-β trigger the phosphorylation of R-Smads that signal trafficking to the nucleus via Smad 4 for specific gene transcription. The phosphorylation of Smad 1/5/8 by BMP7 results in the transcription of target genes that oppose the fibrogenic effects induced by TGF-β-Smad2/3 signaling. BMP7, bone morphogenic protein; TGF-β, transforming growth factor- β1; ECM, extracellular matrix proteins; MMP, matrix metalloproteinase; PAI-1, plasminogen activator inhibitor-1.

Research efforts have determined that a key component of the balance between pro and antifibrotic signaling is the opposing action of BMP7 on TGF-β/Smad signaling (**Figure 1**). It was determined that BMP7 induces the antifibrotic phosphorylation of Smad1/5/8 that opposes TGF-β mediated phosphorylation of Smad2/3 and fibrogenic gene expression (Derynck and Zhang, 2003). Further evaluations into the role of BMP7/Smad signaling in various fibrotic diseases have been performed. In liver disease studies, models of hepato-schistosomiasis and carbon tetrachloride-induced fibrosis have been used to demonstrate the effectiveness of either exogenous BMP7 or adenovirus treatment in reducing key parameters of injury including TGF-β/Smad signaling and hepatic stellate cell activation (Hao et al., 2012; Chen et al., 2013). Additionally, in efforts to define regulators of BMP7/Smad signaling, it was demonstrated that a herbal compound was effective in alleviating hepatic fibrosis via enhancements in p-Smad1/5/8 levels and BMP7 antifibrotic signaling (Hou et al., 2016). In the context of chronic kidney disease, the negative regulation of TGF-β/Smad signaling by BMP7 has been shown to be involved in various nephropathies (Wang et al., 2003; Zeisberg et al., 2003; Chan et al., 2008). Further investigations have detailed parameters involved in the therapeutic restoration mediated by BMP7 overexpression which opposes Smad 3 signaling and protects against TGF-β-induced renal damage (Meng et al., 2013). In other works, the beneficial role of BMP7/Smad signaling has been shown in fibrotic diseases of the lung and heart. Of note, a recent evaluation provided correlative evidence of BMP7 antifibrotic effects in humans compared to animal models of cardiac disease. Particularly, it was determined that left ventricular LV remodeling in patients with aortic stenosis as well as mice with aortic constriction involved impaired BMP/Smad 1/5/8 signaling and increased TGF/Smad2/3 pathways; and that exogenous supplementation with BMP7 reduced LV disease in the mice (Merino et al., 2016). In models of lung disease, similar opposing actions of BMP7 on TGF-β/Smad signaling were indicated by reduced p-Smad 2/3 levels and attenuation of silica-induced pulmonary fibrosis in animals treated with recombinant BMP7 (Yang et al., 2013; Liang et al., 2016). Overall, the importance of BMP7 as an antagonist to profibrogenic TGF-β/Smad signaling has been demonstrated in multiple organ model systems providing support for further investigations into the clinical efficacy of BMP7 treatment strategies.

In addition to affecting Smad-dependent pathways, the antifibrotic role of BMP7 has been linked to several other mechanisms that hold promise for translation into human treatments. For example, DNA methylation changes may be important in BMP7 activity since the prevention of kidney fibrosis was linked to the reversal of Rasal1 promoter hypermethylation (Tampe et al., 2014). In another study, changes in the expression of specific receptors on tubular epithelial cells (e.g., CD44v3) resulted in enhanced BMP7 synthesis and an associated reduction of renal fibrotic damage (Rampanelli et al., 2013). Also, the protective role of BMP7 was shown to be related to changes in the expression of miRNAs as the suppression of miR-21 in rat kidney cells was found to associate with BMP7-mediated inhibition of fibronectin secretion and apoptosis (Yu et al., 2016). Another novel mechanism identified to be associated with BMP7 activity is the production and degradation of hyaluronan in human lung fibroblasts (Midgley et al., 2015). The role of antagonists and competing ligands in the inhibition of BMP7 activity is also part of current investigations which may lead to future translational advancements (Lin et al., 2005; Yanagita et al., 2006; Tanaka et al., 2010). In particular, research is underway to define the role of Activin A, a member of the TGF-β family that acts as an antagonist to BMP7 antifibrotic signaling (Abe et al., 2004; Aykul and Martinez-Hackert, 2016). To date, preclinical studies have shown the potential benefit of inhibiting Activin A signaling by disrupting receptor kinase activity or by blocking the receptor binding site of the antagonistic ligand (Laping et al., 2002; Pearsall et al., 2008; Aykul and Martinez-Hackert, 2016). In addition to the use of antagonists, mechanisms are being studied to enhance antifibrotic signaling through the action of BMP agonists. Notably, a recently developed synthetic peptide (THR-123, Thrasos Therapeutics, Canada) to the BMP receptor activin-like kinase 3 (ALK3), was found to be effective in controlling renal fibrosis in mice (Sugimoto et al., 2012). The successful preclinical work with THR-123 has led to the development of a similar analog, THR-184, which has been tested in a phase 2 trial for the resolution of acute kidney injury in cardiac patients (ClinicalTrials.gov ID NCT01830920). Although it is anticipated that forthcoming results will be clinically revealing, there are limitations in the global use of BMP agonists since the ALK3 receptor is expressed predominantly in the kidney (Sugimoto et al., 2012). Thus, the organ-specificity of BMP agonists needs to be considered and requires further characterization.

Overall, it is well established that BMP7 is an opposing mechanism to TGF-β-mediated profibrogenic signaling and that BMP7 activity is downregulated in fibrotic tissue injury. The potential therapeutic restoration of BMP7 through overexpression or exogenous administration has been demonstrated in multiple animal and organ models of fibrotic disease. However, the effectiveness of these strategies could be limiting due to potential off-target effects and low bioavailability of exogenous BMP7. It is known that systemically administered BMP7 has a short half-life resulting in the need to use of high doses to reach pharmacological effects (Vukicevic et al., 1998). Consequently, the stimulation of BMP signaling can occur in unwanted organ systems due to the ubiquitous expression of BMP receptors throughout the body (Wang et al., 2014). Therefore, current research efforts are focusing on the development and testing of alternative strategies to more specifically target BMP7 including the use of antagonist inhibitors and BMP agonists. Such investigations will likely contribute to translational clinical trials and the advancement of BMP7 as an antifibrotic agent.

## Manipulation of miRNA Expression as an Antifibrotic Strategy

MicroRNAs are short non-coding RNA molecules that regulate target messenger RNAs through post-transcriptional or translational repression mechanisms (Ambros, 2004). It has been shown that miRNA expression levels can be altered in disease states compared to normal conditions, highlighting the potential use of miRNAs as diagnostic or treatment targets. Studies to date have identified unique miRNA profiles for disease states which are often found to be tissue and cellular specific. The dysregulation of miRNA expression during fibrotic disease can involve the aberrant overexpression as well as the downregulation of miRNAs (Bartel, 2009). Regardless of the change, altered miRNA levels can lead to the regulation of a multitude of protein coding genes and signaling mechanisms that promote disease. In fibrosis, miRNAs alterations can lead to disease-promoting changes in a variety of fibrotic mechanisms such as the action of signaling mediators such as TGF-β or the expression of tissue-remodeling ECM components (Jiang et al., 2010). Thus, because of the magnitude of their regulatory role, miRNAs have emerged as viable targets for therapeutic intervention of fibrotic pathways. The following discussion is a review of miRNAs that are promising candidates for the treatment of fibrotic disease.

Research into the role of miRNAs as an antifibrotic therapy in cardiovascular disease is a leading area of investigation. As in other organs, the transformation of cardiac tissue after injury can involve TGF-β signaling and fibroblast activation leading to detrimental scar formation (Khan and Sheppard, 2006). Studies have identified miRNAs in cardiac cells that contribute to the fibrotic cascade in various models of heart disease including atrial fibrosis, cardiac infarction and heart failure as summarized in **Table 2**. Several miRNAs have been noted to be upregulated in animal and human hearts including miR-214, miR-223, and miR-21 (Thum et al., 2008; van Rooij et al., 2008; Roy et al., 2009). Of note, the enhancement of miR-21 led to functional changes in cardiac cells through MAP kinase signaling, fibroblast growth factor 2 expression and the survival of cardiac fibroblasts. Further, a direct link to profibrotic mechanisms was confirmed when the inhibition of miR-21 via antogomir-21 treatments resulted in recovery of heart function in mice subjected to transverse aortic constriction (Thum et al., 2008). In other works, the downregulation of miRNAs was also observed in cardiac disease models including reductions in miR-133, miR-590, miR-30, miR155, miR-22, miR-29, and miR101 (van Rooij et al., 2008; Duisters et al., 2009; Shan et al., 2009; Pan et al., 2012; Kishore et al., 2013; Hong et al., 2016). In recent preclinical assessments, the functional significance of many of the downregulated miRNAs has been described using several models of cardiac fibrotic diseases. In a model of ischemia/reperfusion injury, the dysregulation of miR-29-30-133 were linked to the activation of TGF-β signaling which could be reversed by triiodothyronine (T3) treatment (Nicolini et al., 2015). Particularly, T3 treatment was found to be effective in countering the injury-related downregulation of miR-29c, miR-30c, and miR-133a resulting in the reduction of profibrogenic matrix metalloproteinase (MMP)-2 and CTGF expressions (Nicolini et al., 2015). In models of fibrosis induced by myocardial infarction (MI), the reduction of miRNAs has been shown to regulate many profibrotic proteins and signaling mediators. For example, the targeting of miR-22 shows clinical potential as this miRNA was found to be a negative regulator of cardiac fibrosis through the suppression of TGF-βR1 (Hong et al., 2016). The miR-29 and miR-101 families have also been identified as regulators of antifibrotic mechanisms and have been evaluated for their potential as therapeutic agents. In the case of the miR-29 family, miR-29a, miR-29b, and miR-29c were found to be reduced after MI and associated with the gene expression of ECM proteins and TGF-β signaling (van Rooij et al., 2008; Kriegel et al., 2012). Further, the synthetic overexpression of miR-29 reduced collagen production and fibrosis following MI (van Rooij et al., 2008). Interestingly, the antifibrotic effect of the cardioprotective drug, Tanshinone IIA, was found to involve the upregulation of miR-29b (Yang et al., 2015). Studies evaluating the miR-101 family have similarly demonstrated a protective role in healthy tissue and the importance of designing strategies to increase their expression following cardiac injury. As such, decreased levels of miR-101a/b after coronary artery ligation have been associated with the induction of profibrogenic signaling mediated by c-Fos and TGF (Pan et al., 2012). Importantly, the overexpression of miR-101a inhibited the signaling pathways and alleviated fibrosis in the injured heart. Overall, there is strong preclinical evidence in various injury models that miR-based therapy may be an effective antifibrotic strategy, and especially for the treatment of MI-related cardiac fibrosis. As discussed, the successful modulation of miRNAs by the use of inhibitors, genetic manipulation or pharmacological agents indicates the high therapeutic value of targeting miRNAs. Although the characterization of potential off-target effects of systemically delivered miRNA modulators remains to be determined, the future is bright for the effective and tissue-specific delivery of miRNA targeting treatments.

**Table 2:** Notable miRNAs in cardiac fibrosis; targets and potential therapeutic benefits.

miRNAs	Expression	Model	Target	Effect on cardiac fibrosis	Reference
miR-21	↑	MI Cardiac fibroblasts	PTEN MAPK	↑ MMP2 expression, matrix remodeling, fibroblast survival, interstitial fibrosis	Roy et al., 2009; Thum et al., 2008
miR-29	↓	I/R, MI	TGF-β	↑ MMP2 expression, excessive reparative fibrosis	van Rooij et al., 2008; Kriegel et al., 2012; Nicolini et al., 2015; Yang et al., 2015
miR-30-133	↓	I/R	CTGF	↑ Collagen production	Duisters et al., 2009; Nicolini et al., 2015
miR-22	↓	MI	TGF-βRI	↑ Collagen deposition	Hong et al., 2016
miR-101	↓	MI	c-Fos TGF-β	↑ Collagens, fibronectin, MMP-2, MMP-9	Pan et al., 2012

*MI, myocardial infarction; PTEN, phosphatase and tensin homolog; MAPK, mitogen-activated protein kinase; I/R, ischemia/reperfusion; TGF-β, transforming growth factor beta; CTGF, connective tissue growth factor; TGF-βRI, transforming growth factor beta receptor type I.*

In addition to cardiac disease, the potential of targeting miRNAs in other fibrotic diseases is also of current clinical importance. In the last 10 years, research efforts have increased in the study of miRNAs in hepatic, renal, and pulmonary fibrosis. In liver fibrosis research, the role of hepatic stellate cells (HSCs) in the production of ECM components and fibrotic injury has been well-characterized (Iredale et al., 2013; Lemoinne et al., 2013). As with other fibrotic conditions, several miRNAs were found to be either upregulated or reduced with the altered expression level associating with HSC activation and liver fibrogenesis (Guo et al., 2009). The correlation of miRNA changes to functional outcomes has been shown for several of the miRNAs highlighting their translational potential (Schon et al., 2016). Examples include a noted reduction in HSC-produced collagens in response to the overexpression of miR-133a (Roderburg et al., 2013). In another study, the activation of HSCs was inhibited by treatment with salvianolic acid B and the induced expression of miR-152 (Yu et al., 2015). And as observed in cardiac disease, the downregulation of miR-29 is strongly associated with hepatic fibrosis. Notably, miR-29 mimics or overexpression has been shown to control ECM production by HSCs demonstrating therapeutic potential (Kwiecinski et al., 2011).

The role of miRNAs in renal fibrosis has also been associated with the regulation of signaling pathways that induce ECM factors such as collagens and fibronectin (Wang et al., 2008; Kato et al., 2009). Multiple works have defined the role of miRNAs in renal fibrosis including the effects of miR-148b and members of the miR-29 and miR-let7 families (Fang et al., 2013; Nagai et al., 2014; Szeto and Li, 2014; Srivastava et al., 2016). Additionally, recent studies have demonstrated functional advancements as protection from fibrosis was gained from anti-miR-214 treatment (Denby et al., 2014). Further, the delivery of miR-let7c-expressing mesenchymal stem cells was found to be an effective method to target miRNAs in the damaged kidney (Wang B. et al., 2016). Altogether, research continues to define the therapeutic potential of miRNAs in renal fibrosis.

And lastly, investigation into the role of miRNAs in pulmonary fibrosis is emerging with potential targetable miRNAs identified including miR-29 (Cushing et al., 2015), miR-155 (Pottier et al., 2009), miR-21 (Zhou et al., 2015b; Liu et al., 2016), miR-26a (Liang et al., 2014), and miR-326 (Das et al., 2014). Interestingly, preclinical support of translational effects was indicated by the demonstration of pulmonary fibrosis that is induced as a result of miR-26a inhibition (Liang et al., 2014); and that pulmonary fibrosis could be effectively attenuated following the intranasal deliver of miR-326 mimics (Das et al., 2014). Overall, the increasing and emerging research in this field exemplify the power of targeting miRNAs for the treatment of fibrotic diseases and support the need of future evaluations.

## Peroxisome Proliferator-Activated Receptors

**FIGURE 2:**
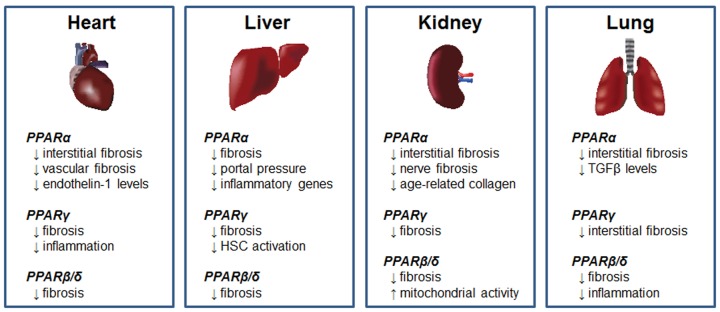
Antifibrotic effects of PPARs. Summary of the antifibrotic effects of PPARα, PPARγ, and PPARβ/δ agonists in models of cardiac, hepatic, renal and pulmonary fibrosis.

The PPARs are nuclear transcription factors that form obligate heterodimers with retinoid-X receptors to modulate transcription of target genes. Three PPAR subtypes have been identified, known as PPARα, PPARβ/δ, and PPARγ. Due to its role in the insulin-sensitizing effects of the thiazolidinedione (TZD) drugs, PPARγ is by far the most widely studied PPAR isoform. However, preclinical studies have implicated all three PPARs as potential targets for antifibrotic therapy (summarized in **Figure 2**). Recent studies supporting the use of PPAR agonists for this purpose are described below, along with discussion of recently completed or ongoing clinical trials.

### PPARγ Agonists

Peroxisome proliferator-activated receptorsγ is involved in insulin sensitization and the promotion of adipogenesis (Gross et al., 2017). It is targeted primarily as a treatment for diabetes, where it enhances the insulin sensitivity of target tissues to promote glucose uptake. It also has potent lipid-lowering properties, and is anti-inflammatory. The endogenous ligands for PPARγ are thought to be prostaglandin and leukotriene derivatives, including 15-deoxy-Δ-12,14-prostaglandin J2 (15d-PJ2). Synthetic ligands include the TZD drugs, such as rosiglitazone and pioglitazone, used clinically for diabetes treatment. The TZD have also shown promise in preclinical studies of established fibrosis, as well as in a limited number of clinical studies (summarized in **Table 3**).

**Table 3:** Selected preclinical and clinical studies using PPARγ agonists for established fibrosis.

Preclinical studies in animal models with established fibrosis					
**Fibrotic disease**		**Species**	**Model**	**Drug**	**Findings**	**Reference**

Cardiac fibrosis		Mouse	AT-II	PGZ	Decreased fibrosis and inflammation	Caglayan et al., 2008
		Rat	Pressure overload	RGZ	Reduced fibrosis	Qi et al., 2015
		Rat	MCT	PGZ	Reduced right ventricular fibrosis and cardiomyocyte size	Behringer et al., 2016
		Mouse	T1DM (Akita)	CGZ	Reduced fibrosis, improved end diastolic diameter	Mishra et al., 2010
		Rat	T2DM (OLETF)	PGZ	Reduced fibrosis, increased MMP9 expression	Makino et al., 2009; Ihm et al., 2010
Hepatic fibrosis		Rat	CCl_4_, BDL, CDAA	PGZ	Ineffective after fibrosis was established	Leclercq et al., 2006
		Mouse	CCl_4_	PGZ	Ineffective after disease was established	Da Silva Morais et al., 2007

**Clinical studies of fibrosis**						

**Disease (*n)***	**Study type**	**Drug**	**Dose**	**Duration**	**Findings**	**Reference**
NASH *(25)*	P	RGZ	4 mg/day	48 weeks	Improved steatosis, inflammation, and fibrosis	Neuschwander-Tetri et al., 2003
NASH *(18)*	P	PGZ	30 mg/day	48 weeks	Improved steatosis, inflammation, and fibrosis	Promrat et al., 2004
NASH *(61)*	R,PC,DB	PGZ	30 mg/day	12 months	Improved steatosis, inflammation, and fibrosis	Aithal et al., 2008
NASH *(108)*	R,P,OL	RGZ	4 mg 2x/day	12 months	Improved steatosis, inflammation, and fibrosis	Torres et al., 2011
NASH *(63)*	R,PC,DB	RGZ	8 mg/day	12 months	Improved steatosis and liver function, no effect on fibrosis	Ratziu et al., 2008
NASH *(142)*	R,PC,DB	PGZ	30 mg/day	96 weeks	Improved steatosis and inflammation, no effect on fibrosis	Sanyal et al., 2010

*AT-II, Angiotensin-II; BDL, bile duct ligation; CDAA, choline deficient L-amino acid defined diet; CGZ, ciglitazone; DB, double-blind; MCT, monocrotaline; n: number of patients who completed the study with pre- and post-treatment biopsies; NASH, non-alcoholic steatohepatitis; P, prospective; PC, placebo-controlled; PGZ, pioglitazone; R, Randomized; RGZ, rosiglitazone; T1DM, Type 1 diabetes mellitus; T2DM, Type 2 diabetes mellitus; OLETF, Otsuka Long-Evans Tokushima rats*

A role for PPARγ in the control of fibrosis has long been apparent, with many of the studies focused on the liver. Early studies of liver myofibroblasts (hepatic stellate cells, or HSCs) revealed that PPARγ expression was present in quiescent HSC, but was reduced with activation to the myofibroblastic phenotype and fibrosis progression (Galli et al., 2000; Marra et al., 2000; Miyahara et al., 2000). Overexpression of PPARγ in HSC, or treatment with the endogenous PPARγ ligand 15d-PGJ2 or TZDs, resulted in decreased myofibroblastic character of HSCs, with reduced collagen production and increased MMP activity (Galli et al., 2000; Marra et al., 2000; Miyahara et al., 2000; Hazra et al., 2004). However, targeting PPARγ in preclinical animal models of liver fibrosis by treatment with TZDs has met with mixed results. In preclinical studies in rats, TZD treatment prevented acute CCl_4_-induced liver damage (Kon et al., 2002), and chronic fibrosis induced by toxins, cholestasis, or choline-deficient diet (Galli et al., 2002; Kawaguchi et al., 2004; Marra et al., 2005; Bruck et al., 2009). Furthermore, recent studies using the endogenous PPARγ ligand 15d-PGJ2 prevented hepatic fibrosis induced by *Trypanosoma cruzi* infection and carbon-tetrachloride-induced fibrosis in mice (Jia et al., 2015; Penas et al., 2016). However, studies using PPARγ agonists in treatment models of established liver disease have met with very different results. In rat models of fibrosis, pioglitazone prevented toxin (CCl_4_) and choline-deficient diet fibrosis, but was not effective when administered after the disease was established, and was ineffective against cholestasis-induced injury regardless of the length of treatment (Leclercq et al., 2006). Furthermore, pioglitazone was ineffective at reducing the activation of mouse HSC, and failed to prevent CCl_4_-induced hepatic fibrosis (Da Silva Morais et al., 2007).

The reason for the discrepancies in the preclinical models is not currently known, but it has been speculated that effects of thiazolidinediones on other targets, such as alterations in adiponectin signaling or promotion of the fibropermissive (M2) phenotype of macrophages, may have counteracted the antifibrotic effects (Da Silva Morais et al., 2007; Sica et al., 2014). Furthermore, although long-term (12 months) treatment of NASH patients with rosiglitazone was associated with reduced liver fibrosis, there was evidence of increased liver inflammation (Lemoine et al., 2014), while similar studies using pioglitazone generally showed a decrease in inflammation (Mahady et al., 2011). The reason for this observation is not clear, but may be due to promotion of inflammatory cytokines by hepatocytes in response to rosiglitazone (Rogue et al., 2011; Lemoine et al., 2014). To circumvent these problems, targeted delivery of PPARγ agonists may be more efficacious. In support of this notion, nanoformulation of rosiglitazone into a biodegradable copolymer effectively reduced common bile duct ligation-induced hepatic fibrosis when administered early in the development of the disease (Kumar et al., 2014). Similarly, rosiglitazone packaged into liposomes with a HSC-targeting moiety effectively decreased established hepatic fibrosis in rats (Patel et al., 2012). Other approaches include the use of agents that activate PPARγ by alternate pathways. Recent studies suggested that treatment with rosmarinic acid and baicalin reduced hepatic fibrosis, by de-repression of PPARγ gene expression by the protein MeCP2, a process that could be reversed by inhibiting the Wnt signaling pathway (Yang et al., 2012; Kweon et al., 2016). Finally, it is possible that new, non-TZD activators of PPARγ, may prove more effective and with fewer side effects than TZDs. In support, a recent study showed that the non-TZD agonist GW570 prevented both cholestasis and CCl_4_ induced fibrosis (Yang et al., 2010).

Peroxisome proliferator-activated receptorsγ also has a role in the progression and treatment of cardiac fibrosis. Many of the preclinical studies have focused on cardiac damage in association with rodent hypertensive models. Pioglitazone decreased cardiac fibrosis in spontaneously hypertensive rats (Nakamura et al., 2008). In rat pressure overload models, rosiglitazone prevented cardiac fibrosis, while a PPARγ antagonist worsened fibrosis (Gong et al., 2011). Using a similar model, ciglitazone prevented fibrosis, and promoted the expression of matrix-degrading enzymes (Henderson et al., 2007). In a mouse angiotensin-II treatment model, pioglitazone reduced cardiac fibrosis (Caglayan et al., 2008). Interestingly, this study used either macrophage- or cardiomyocyte-specific knockout of PPARγ, and concluded that PPARγ in both cell types contributed to decreased macrophage infiltration. However, pioglitazone decreased cardiomyocyte fibrosis and inflammation both wild-type and cardiomyocyte PPARγ-knockout mice, but not in macrophage PPARγ-knockout mice, suggesting that in this model, macrophage PPARγ activity is critical for the antifibrotic effect of pioglitazone. Treatment of established pressure overload-induced cardiac fibrosis with rosiglitazone reduced interstitial cardiac fibrosis and fibrosis-related gene expression (Qi et al., 2015). Using the cardiac pressure overload model in rats, TSG (2,3,4′,5-tetrahydroxystilibene-2-*O*-β-d-glucoside) administered early in the progression of the disease (3 days post-surgery) reduced cardiac fibrosis and expression of types I and II collagen through a pathway involving PPARγ (Peng et al., 2016). In a treatment model of established pulmonary artery hypertension-induced right ventricular cardiac fibrosis, pioglitazone treatment reduced right ventricular fibrosis and cardiomyocyte size (Behringer et al., 2016). The lipid-lowering drug atorvastatin prevented cardiac fibrosis induced by advanced glycation end-products (AGEs), by a mechanism that appeared to involve PPARγ (Chen et al., 2016). Similar findings were reported in a toxin-induced model of pulmonary fibrosis, where atorvastatin was found to be more effective that pioglitazone (Malekinejad et al., 2013). However, it is currently unclear whether atorvastatin directly or indirectly activates PPARγ.

Positive results have also been observed in models of diabetes-related cardiac fibrosis. In alloxan-induced diabetic rabbits, rosiglitazone prevented atrial remodeling and fibrosis (Liu et al., 2014). Similarly, ciglitazone treatment reduced cardiac fibrosis and improved end diastolic diameter in diabetic Akita mice (Mishra et al., 2010), while rosiglitazone reduced myocardial fibrosis in diabetic Otsuka Long-Evans rats (Makino et al., 2009; Ihm et al., 2010).

Finally, PPARγ has also been a target for treatment of fibrosis in several other organs. In relation to pulmonary fibrosis, bleomycin-induced lung injury in rodents was prevented by TZD or 15d-PGJ2 treatment (Genovese et al., 2005; Milam et al., 2008; Aoki et al., 2009; Samah et al., 2012). In a recent study, rosiglitazone prevented pulmonary fibrosis induced by radiation exposure (Mangoni et al., 2015). In a bleomycin-induced model of dermal fibrosis, rosiglitazone reduced inflammation and collagen deposition (Wu et al., 2009). The new PPARγ agonist GED-0507-34-Levo, when administered early in the progression of chronic colitis in mice, reduced intestinal fibrosis (Speca et al., 2016). This compound is currently in a phase 2 clinical trial as a treatment for ulcerative colitis (ClinicalTrials.gov ID NCT02808390). Finally, PPARγ has also been an effective treatment for some models of nephropathy-related fibrosis (Jia et al., 2014; Speeckaert et al., 2014).

Clinical trials assessing the treatment of fibrotic diseases with PPARγ agonists have largely been limited to patients with non-alcoholic steatohepatitis (NASH), and the results have been decidedly mixed. Early prospective studies of patients with NASH before and after 48 weeks treatment with either rosiglitazone or pioglitazone showed significant improvements in steatosis, inflammation and fibrosis (Neuschwander-Tetri et al., 2003; Promrat et al., 2004). Similar results were observed in prospective 12 months studies of rosiglitazone or pioglitazone treatment of NASH patients (Aithal et al., 2008; Torres et al., 2011). Conversely, a prospective 12 months study using rosiglitazone found no effect on fibrosis (Ratziu et al., 2008). A relatively short-term study (26 weeks) of insulin-resistant patients with NASH treated with pioglitazone or placebo in conjunction with a hypocaloric diet showed improvements in liver function, steatosis and inflammation, but no difference in fibrosis compared with placebo (Belfort et al., 2006). A phase 3 randomized, multi-center placebo-controlled trial that was conducted to determine the effect of 96 weeks treatment of pioglitazone, vitamin E or placebo on patients with NASH revealed that, while pioglitazone treatment resulted in significantly improved liver function, steatosis, and inflammation, there was no significant effect on fibrosis (Sanyal et al., 2010). The reasons for the discrepancies in these studies are not clear, but may involve differences in patient population (e.g., inclusion or exclusion of diabetes patients, differences in medication) and study design between the individual trials. There may also be a difference in the relative effectiveness between thiazolidinedione, as a recent meta-analysis has concluded that pioglitazone, but not rosiglitazone, significantly decreased fibrosis in NASH (Musso et al., 2017). A phase 2 trial is currently underway to study pioglitazone in patients with non-alcoholic steatohepatitis, which includes liver inflammation and fibrosis as a secondary aim (ClinicalTrials.gov ID NCT01068444).

The use of the TZD drugs has waned considerably in recent years due to side effects such as weight gain, edema and bone density loss (Tahrani et al., 2016), as well as recent concerns regarding rosiglitazone and pioglitazone and the increased risk of cardiovascular disease and bladder cancer, respectively, although the latter associations remain controversial (Hoogwerf et al., 2016; Hampp and Pippins, 2017). Recent studies have focused on PPARγ partial agonists, also known as selective PPARγ modulators (SPPARMs), which retain the insulin-sensitizing effects of PPARγ activation, but lack the strong adipogenic effects. One of these synthetic PPARγ agonists, bardoxolone (also known as CDDO) effectively reduced collagen and antagonized TGF-β signaling in two models of dermal fibrosis, but the antifibrotic effects appeared to be independent from PPARγ (Wei et al., 2013). Another, INT131, showed promising results in reducing blood glucose in type 2 diabetes patients without weight gain or fluid retention (Dunn et al., 2011). To date, clinical studies of non-TZD PPARγ agonists on fibrotic diseases are lacking.

### PPARα Agonists

Peroxisome proliferator-activated receptorsα is predominantly expressed in hepatocytes, cardiomyocytes, renal proximal tubule cells, and enterocytes. The main function of PPARα is to promote lipid β-oxidation, partially through promotion of peroxisomal enzymes, and thus is a target of lipid-lowering drugs such as the fibrates, the best known of which is fenofibrate. The endogenous ligands of PPARα are thought to be lipids, including fatty acids and arachidonic acid derivatives.

It has been long known that PPARα plays a role in tissue fibrosis. PPARα-null mice developed age-related myocardial fibrosis, which was detectable at 16 weeks of age, and pronounced by 32 weeks (Watanabe et al., 2000). In several different models of cardiac fibrosis, fenofibrate prevented interstitial and perivascular cardiac fibrosis, through a mechanism that involved reduced endothelin-1 levels (Ogata et al., 2002, 2004; Iglarz et al., 2003; Diep et al., 2004; LeBrasseur et al., 2007; Forcheron et al., 2009; Zhang et al., 2016). Similar results were observed using inducible overexpression of PPARα early in the progression of pressure overload-induced cardiac damage (Kaimoto et al., 2016). However, one study using fenofibrate treatment of chronic pressure overload in PPARα knockout mice showed that fenofibrate had profibrotic effects that were independent of PPARα (Duhaney et al., 2007).

Fenofibrate prevented interstitial renal fibrosis in a number of preclinical rodent models (Hou et al., 2010; Li et al., 2010; Tanaka et al., 2011; Balakumar et al., 2014). Similar results were observed using other PPARα agonists, gemfibrozil and BAY PP1 (Calkin et al., 2006; Boor et al., 2011). Fenofibrate prevented renal nerve fibrosis and injury in a type 2 diabetes mouse model (Cho et al., 2014), renal fibrosis in a rat type 1 diabetes model (Cheng et al., 2016), and age-related renal collagen accumulation in mice (Kim et al., 2016). In addition to the kidney, fenofibrate preventively decreased bleomycin-induced pulmonary fibrosis, and decreased lung TGFβ content (Samah et al., 2012).

In the liver, the strong PPARα agonist WY-14643 reduced fibrosis induced by a choline-deficient diet (Ip et al., 2004), and decreased fibrosis induced by a combination of ethanol feeding and CCl_4_ injections (Nan et al., 2013). In a treatment model of established cirrhosis in rats, fenofibrate reduced portal pressure and fibrosis (Rodríguez-Vilarrupla et al., 2012). Fenofibrate prevented hepatic fibrosis induced by thioacetamide or concanavalin-A (Toyama et al., 2004; Mohamed et al., 2013). Interestingly, the ability of fenofibrate to attenuate hepatic steatosis and fibroses appears to be associated with its trans-repressive effects on inflammation- and fibrosis-related genes, rather than its ability to transcriptionally regulate genes associated with lipid metabolism (Pawlak et al., 2014).

Few clinical studies of PPARα agonists in fibrotic diseases have been conducted, and most of these focused on primary biliary cirrhosis, due largely to the lipid and bile acid modulating effects of the fibrate drugs, and their anti-inflammatory properties. However, most studies did not assess the effect on liver fibrosis directly. One study did show that fenofibrate reduced liver stiffness and serum hyaluronic acid, a marker of ECM accumulation (El-Haggar and Mostafa, 2015).

### PPARβ/δ Agonists

Peroxisome proliferator-activated receptorsβ/δ, also known as PPARβ or PPARδ, is ubiquitously expressed. The physiological function of PPARβ/δ is not clear, but activation of the receptor results in modulation of lipid and glucose homeostasis, skeletal muscle function, and brown adipose tissue activity (Gross et al., 2017). The endogenous ligands for PPARβ/δ are thought to be lipid derivatives, and synthetic agonists have been produced for preclinical studies.

Treatment of fibrotic diseases with agonists of PPARβ/δ has been limited to preclinical animal studies. One synthetic agonist, GW0742, reduced bleomycin-induced lung fibrosis and inflammation in mice (Galuppo et al., 2010), and prevented pulmonary artery banding-induced cardiac hypertrophy and fibrosis in mice (Kojonazarov et al., 2013). In a rat model of type-1 diabetes-associated cardiac fibrosis, GW0742 reduced markers of cardiac fibrosis (Chang et al., 2016).

The PPARβ/δ agonist GW610742 was effective in preventing collagen content in a rat corneal wounding model (Gu et al., 2014), while another agonist (GW501516) reduced peritoneal fibrosis and inflammation in rats (Su et al., 2014). However, in a rat model of MI, administration of GW610742 resulted in an early increase in cardiac fibrosis (7 days after MI), although there was no overall effect on collagen levels after 14 days (Park et al., 2016). Another PPARβ/δ agonist, HPP593, was effective in reducing renal fibrosis induced by chronic ischemia, by a mechanism that appeared to be due, at least in part, by a reduction in oxidative stress and preservation of mitochondrial function (Fedorova et al., 2013).

One study using the synthetic agonist KD3010 observed effective prevention of liver fibrosis induced by CCl_4_ or chronic cholestasis (Iwaisako et al., 2012). Interestingly, the same study showed that GW501516 had no effect on fibrosis in the same models. One possible explanation is the observation that GW501516, but not KD3010, induced hepatic connective tissue growth factor, a profibrotic factor. Consistent with this observation, another study showed that GW501516, administered to mice concomitantly with CCl_4_, enhanced the degree of fibrosis and inflammation, by a mechanism that involved enhanced proliferation of the hepatic stellate cells (Kostadinova et al., 2012).

### Dual-, Pan-, and Mixed-PPAR Agonists

Many studies have attempted to use multi-specificity PPAR agonists, to varying degrees of success (Derosa et al., 2017). In the following section, recent studies using dual-, pan-, or mixed PPAR agonists will be discussed in terms of their potential for future treatment of fibrotic disease.

#### Dual-PPAR Agonists

A number of dual PPARα/γ activators, collectively known as glitazars, have been produced. One of these, tesaglitazar, reduced diabetic nephropathy in obese diabetic *db/db* mice (Cha et al., 2007). Similarly, another dual PPARα/γ activator, aleglitazar, was effective in promoting glucose regulation and renal function, and decreasing pancreatic islet fibrosis and degeneration in obese diabetic rats (Bénardeau et al., 2013). A clinical trial has been completed comparing saroglitazar with pioglitazone in the treatment of non-alcoholic fatty liver disease, but the results have yet to be published (ClinicalTrials.gov ID NCT02265276).

Elafibrinor (also known as GFT505), a recently developed dual PPARα/δ activator, was used in multiple rodent models of metabolic and fibrotic liver diseases. The effects were positive in all models, with elafibrinor reducing steatosis, inflammation and fibrosis in both preventative and treatment regimens (Staels et al., 2013). A placebo-controlled clinical study was recently performed with 1 year of elafibranor treatment of NASH patients (Ratziu et al., 2016). Elafibranor induced resolution of steatosis, and improved the fibrosis score in patients with decreased steatosis. A phase 3 trial is underway to determine the effect of elafibranor on NASH patients, with fibrosis evaluation included in the outcomes (ClinicalTrials.gov ID NCT02704403).

#### Pan-PPAR Agonists

A recently developed agonist that weakly activates all three PPARs (IVA337) was used in a mouse model of dermal fibrosis (Ruzehaji et al., 2016). Importantly, the drug was used in both prevention and treatment models, and furthermore was directly compared to the PPARγ agonist rosiglitazone. A phase 2 proof-of-concept study is underway to study IVA337 as a treatment for diffuse scleroderma (ClinicalTrials.gov ID NCT02503644), and a phase 2b trial for the treatment of NASH (ClinicalTrials.gov ID NCT03008070).

#### Mixed PPAR Agonists

Angiotensin 1 receptor antagonists, collectively knowns as the sartans, have long been used to modulate the activity of the renin-angiotensin system. Recently, it was discovered that several members of the sartans have weak PPARγ-activating properties (Michel et al., 2013). Of these, telmisartan appears to be the most potent at activating PPARγ. Telmisartan reduced fibrosis in response to MI in rats (Maejima et al., 2011; Nagashima et al., 2012). Similarly, irbesartan protected against cardiac and renal fibrosis and myocyte hypertrophy in a mouse model of salt-sensitive hypertension (Kusunoki et al., 2013). Interestingly, in both of these cases, most of the antifibrotic effects appeared to be independent of angiotensin receptor blockade, as they were blocked by PPARγ inhibition. Another study observed reduced myocardial fibrosis in angiotensin converting enzyme-2 null mice treated with irbesartan (Zhang et al., 2013). Similarly, irbesartan reduced bleomycin-induce pulmonary fibrosis and inflammation in mice (Tanaka et al., 2013). In a study supporting the concept of dual angiotensin 1 receptor blockage and PPARγ activation, candesartan combined with pioglitazone had increased efficacy in reducing fibrosis in spontaneous hypertensive rats compared with either agent alone (Nakamura et al., 2008). Interestingly, recent studies showed that telmisartan and elmisartan reduced cardiac fibrosis though a mechanism that involved activation of PPARβ/δ (Chang et al., 2016; Wei-Ting et al., 2016), suggesting that PPARγ may not be the only nuclear receptor activated by these agents.

Several cannabinoid compounds have been shown to have dual activity at the CB2 cannabinoid receptor as well as PPARγ and, in some cases, other PPAR isoforms (O’Sullivan, 2007). One of these, ajulemic acid, was effective in preventing and treating bleomycin-induced lung and skin fibrosis (Garcia-Gonzalez et al., 2012; Lucattelli et al., 2016). A new compound (VCE-004.8) that serves as a dual-agonist of PPARγ and the cannabinoid 2 receptor, reduced collagen deposition and dermal thickness in a mouse model of scleroderma (del Rio et al., 2016).

The cannabinoid oleoylethanolamide (OEA) is thought to be an endogenous PPARα agonist, although it can signal through other receptors (De Petrocellis and Di Marzo, 2010). When used to treat mice concurrently with a methionine-choline deficient diet to induce hepatic steatosis and mild fibrosis, OEA was found to reduce overall liver collagen content and decrease the gene expression of collagens type I and III, α-smooth muscle actin (SMA), TIMP1, MMP2, and MMP9 (Chen et al., 2015). Similar results were observed when more extensive hepatic fibrosis was induced using thioacetamide. Importantly, the protective effect of OEA was greatly reduced in PPARα-null mice, suggesting that the beneficial effects were primarily mediated via PPARα, although a role for other OEA receptors cannot be totally excluded.

## Relaxin

**FIGURE 3:**
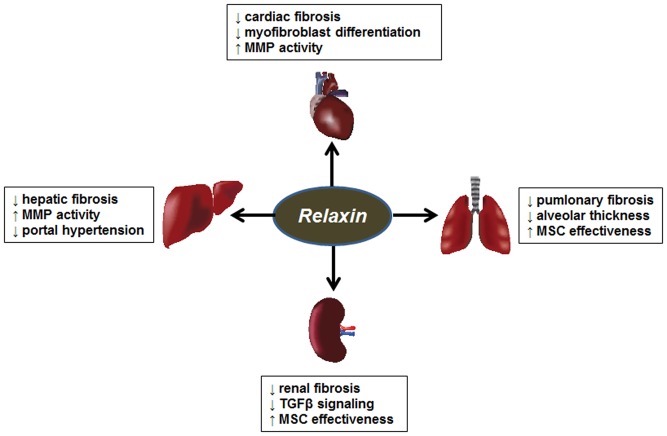
Antifibrotic effects of relaxin. Summary of the antifibrotic effects of PPARα, PPARγ, and PPARβ/δ agonists in models of cardiac, hepatic, renal and pulmonary fibrosis. MMP, matrix metalloproteinase; MSC, mesenchymal stem cells.

Relaxin is a polypeptide of the insulin/relaxin superfamily. It is produced by the corpus luteum of the ovary during pregnancy (Sherwood, 2004; Bathgate et al., 2013). Its roles during pregnancy involve widespread hemodynamic changes such as vasodilation, decreased systemic vascular resistance, and increased renal plasma flow and glomerular filtration rate (Conrad and Davison, 2014). Its roles outside of pregnancy were less clear until the generation of the relaxin knockout mouse, which developed widespread fibrosis with aging (Samuel et al., 2016a). Importantly, the male mice developed fibrosis as well, but the source of relaxin in males and non-pregnant females is unclear. Relaxin protects against fibrosis both by decreasing collagen production, and promoting collagen degradation by increasing the levels and activity of MMPs (Samuel et al., 2016b; Bennett, 2009). Furthermore, many of relaxin’s effects involve antagonism of the effects of TGFβ. The relatively recent discovery of its receptor (RXFP1) has led to rapid development of new discoveries for the use of relaxin as an antifibrotic agent (Bathgate et al., 2013). Some of these findings are summarized in **Figure 3**.

The earliest human studies using relaxin to target fibrotic diseases began in the late 1950s, using partially purified porcine relaxin to treat scleroderma, with variable success (Samuel et al., 2016a). Preclinical animal studies have supported the use of relaxin for dermal fibrosis, (Unemori et al., 1993), and the relaxin knockout mouse spontaneously develops age-related skin fibrosis (Samuel et al., 2005). A peptide activator of RXFP1 prevented bleomycin-induced dermal fibrosis in mice (Pini et al., 2010). Finally, the development of recombinant human relaxin led to more recent clinical scleroderma trials. In a phase 2 study, beneficial effects of relaxin were observed in patients with moderate to severe diffuse systemic sclerosis after 24 weeks infusion with low relaxin (25 μg/kg/day), but not at a higher dose (100 μg/kg/day) (Seibold et al., 2000). However, in a subsequent phase 3 trial, relaxin had no clinically significant effects (Khanna et al., 2009). Part of the lack of success of this study may have been due to the relatively advanced degree of scleroderma in the patient population, a notion supported by the finding that relaxin treatment failed to reverse advanced scleroderma in the relaxin knockout mouse (Samuel et al., 2005), or the uncertainty in clinically meaningful outcomes measures in human systemic sclerosis trials (Seibold, 2002).

Relaxin has been extensively studied as a treatment for several preclinical fibrosis models (summarized in **Table 4**), including cardiac fibrosis. The relaxin knockout mouse serves as a model of age-related cardiac fibrosis and associated left ventricular dysfunction, but interestingly, only in the male mice, due to additional detrimental effects of testosterone (Du et al., 2003; Hewitson et al., 2012). The increased left ventricular collagen deposition was reversed by treatment with relaxin, by a mechanism that appeared to involve inhibition of cardiac myofibroblast differentiation (Samuel et al., 2004a). In preclinical animal models, relaxin treatment, or relaxin delivered by adenovirus, effectively reduced cardiac fibrosis induced by β-adrenergic stimulation in rodents (Samuel et al., 2004a; Zhang et al., 2005; Bathgate et al., 2008). More recently, relaxin was more effective than the angiotensin converting enzyme inhibitor enalapril in treating isoproterenol-induced cardiac injury, while the combination of relaxin and enalapril was more efficacious than either treatment alone, in both prevention and treatment approaches (Samuel et al., 2014). Furthermore, the effect of relaxin treatment on isoproterenol-induced cardiac fibrosis may involve inhibition of endothelial to mesenchymal transition (Zhou et al., 2015a). Relaxin was also effective in reversing cardiac fibrosis in the spontaneously hypertensive rat model (Lekgabe et al., 2005). Furthermore, relaxin reduced both fibrosis and atrial fibrillation in spontaneously hypertensive rats (Parikh et al., 2013). Relaxin reduced collagen content in a rat model of angiotensin II-induced fibrosis, and furthermore, berberine was used to induce relaxin expression, with similar results (Gu et al., 2012). Relaxin was also successful in reducing collagen in a diabetic cardiomyopathy model (Samuel et al., 2008). Conversely, in a chronic pressure overload model, relaxin knockout mice had no more collagen deposition in the heart than wild-type mice (Xu et al., 2008), suggesting that the effectiveness of relaxin may depend on the nature of cardiac injury.

**Table 4:** Selected preclinical studies using relaxin for established fibrosis.

Preclinical studies in animal models with established fibrosis						
**Fibrotic disease**	**Species**	**Model**	**Dose**	**Findings**	**Reference**
Cardiac fibrosis	Mouse	TG- β2AR, Rln-KO	0.5 mg/kg/day	Decreased left ventricular fibrosis, inhibition of cardiac myofibroblast differentiation	Samuel et al., 2004a
	Mouse	TG-β2AR	Adenovirus-delivered	Decreased left ventricular collagen content	Bathgate et al., 2008
	Mouse	Iso-prenaline	0.5 mg/kg/day	Decreased cardiac fibrosis, suppressed TGFβ expression and signaling, increased MMP13	Samuel et al., 2014
	Rat	SHR	0.5 mg/kg/day	Decreased cardiac and renal collagen, suppressed myofibroblast differentiation, increased MM2	Lekgabe et al., 2005
	Rat	SHR	0.4 mg/kg/day	Suppressed atrial fibrillation, decreased fibrosis and hypertrophy	Parikh et al., 2013
	Mouse	STZ-mRen2	0.5 mg/kg/day	Decreased fibrosis and left ventricular stiffness, increased MMP13 and reduced TIMP1	Samuel et al., 2008
	Rat	MI	1 μg/day	Decreased fibrosis and myocardial apoptosis	Bonacchi et al., 2009
Hepatic fibrosis	Mouse	CCl_4_	25–75 μg/kg/day	Reduced fibrosis, increased MMP13 expression, increased collagen degrading activity	Bennett et al., 2014
	Mouse	CCl_4_, BDL	0.5 mg/kg/day	Reduced markers of fibrosis, reduced portal pressure	Fallowfield et al., 2014
Pulmonary fibrosis	Mouse	Rln-KO	0.5 mg/kg/day	Reduced lung collagen content and restored alveolar structure	Samuel et al., 2003
	Mouse	OVA-AAD	0.5 mg/kg/day	Decreased collagen deposition and epithelial thickening, but no effect on inflammation	Royce et al., 2009; Royce et al., 2015
	Mouse	OVA-AAD	0.8 mg/ml IN	Decreased lung collagen and epithelial thickening	Royce et al., 2014
Renal fibrosis	Mouse	Rln-KO	0.5 mg/kg/day	Decreased kidney collagen	Samuel et al., 2004b
	Rat	Aging	96 μg/day	Reduced renal collagen, improved renal function	Danielson et al., 2006
	Rat	DS	12 μg/day	Decreased collagen and TGFβ signaling, improved systolic blood pressure	Yoshida et al., 2011
	Mouse	UUO	0.5 mg/kg/day	Reduced renal collagen and myofibroblast differentiation	Huuskes et al., 2015
	Mouse	STZ-eNOS-KO	32 or 320 μg/kg/day	No effect on renal fibrosis	Dschietzig et al., 2015

*DS, salt-sensitive rat model; IN, intranasal; MI, myocardial infarction; MMP, matrix metalloproteinase; OVA-AAD, ovalbumin-induced allergic airway disease; Rln-KO, Relaxin knockout mouse; SHR, spontaneously hypertensive rats; STZ-mRen2 (streptozotocin-treated mRen2 transgenic mice); STZ-eNOS-KO, streptozotocin-treated eNOS knockout mice; TG-β2AR, transgenic β2-adrenergic receptor overexpressing mice; TIMP, tissue inhibitor of metalloproteinase; UUO, unilateral ureteral obstruction*

In rats subjected to MI, relaxin reduced cardiac fibrosis, inhibited cardiac myofibroblast differentiation, and promoted induction of MMPs (Bonacchi et al., 2009; Samuel et al., 2011). Furthermore, a recent study suggested that in addition to reduced post-MI-induced fibrosis, relaxin reduced tachyarrhythmia and cardiac dysfunction in rats (Wang D. et al., 2016). Recombinant human relaxin has been used in clinical studies of acute heart failure, with some promising results in regard to dyspnea and hemodynamic properties, as well as cardiovascular death and all-cause mortality in a phase III study (Teerlink et al., 2009). A second phase III trial is currently underway to study the effect of relaxin on acute heart failure (ClinicalTrials.gov ID#NCT02064868).

The fibrotic lung is also a therapeutic target of relaxin. The relaxin knockout mouse developed age-related pulmonary fibrosis, and as with cardiac fibrosis, the effect was more pronounced in male mice, and was reversed after relaxin treatment (Samuel et al., 2003). Similarly, the relaxin knockout mouse developed more severe fibrosis in studies using ovalbumin-induced allergic airway disease (Mookerjee et al., 2006; Samuel et al., 2007). In rodent models of pulmonary fibrosis induced by bleomycin or ovalbumin-induced allergic airway disease, lung collagen content and alveolar thickness were reduced with relaxin (Unemori et al., 1996; Royce et al., 2009). Recent studies also showed efficacy of intranasal relaxin administration in allergic airway disease and cigarette smoke induced lung damage (Royce et al., 2014; Pini et al., 2016). In an exciting development, relaxin and bone marrow-derived mesenchymal stem cells or amnionic epithelial stem cells were found to be synergistic in the treatment of established airway disease (Royce et al., 2015, 2016). The mechanism for this effect is currently under investigation, but appears to be related to the matrix remodeling and anti-TGFβ effects of relaxin promoting a favorable environment for the establishment of stem cell residency in the damaged tissue (Samuel et al., 2016b).

Treatment of experimental renal fibrosis has also supported relaxin as a novel antifibrotic agent. As with the heart, skin and lungs, the relaxin knockout mouse develops renal fibrosis with age, which can be reversed with relaxin treatment (Samuel et al., 2004b). In several animal models of renal fibrosis, relaxin was effective in reducing collagen deposition (Garber et al., 2001, 2003; McDonald et al., 2003; Lekgabe et al., 2005; Danielson et al., 2006; Yoshida et al., 2011; Yoshida et al., 2014). Relaxin reduced unilateral uteretic obstruction-induced renal disease by antagonizing the effect of TGF-β, and that this effect was enhanced by coadministration of mesenchymal stem cells (Hewitson et al., 2010; Huuskes et al., 2015). On the other hand, relaxin was not effective in a diabetes-related renal disease model (Wong et al., 2013; Dschietzig et al., 2015). However, unlike the unilateral uteretic obstruction models, TGFβ was not increased in the diabetic model. Therefore, given relaxin’s well-known role in opposing the profibrotic effects of TGFβ, the lack of effect in the diabetes model is perhaps not surprising.

There is also evidence that relaxin can treat liver fibrosis. In prevention models, relaxin reduced collagen production and promoted MMP in CCl_4_-induced hepatic fibrosis (Williams et al., 2001; Bennett et al., 2009). In a more clinically relevant model, relaxin treatment also reduced established liver fibrosis (Bennett et al., 2014). Finally, relaxin reduced portal hypertension in animal models of liver disease (Fallowfield et al., 2014), and a phase 2 trial is currently underway to study the effect of relaxin on portal hypertension in cirrhotic patients (ClinicalTrials.gov ID# NCT02669875).

**FIGURE 4:**
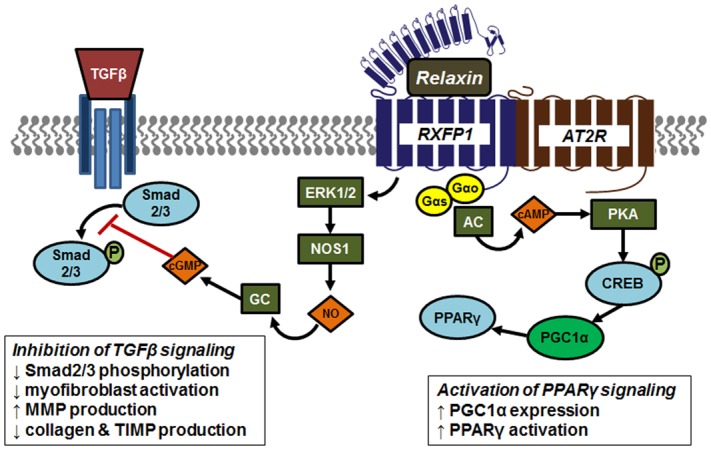
Signaling and crosstalk associated with relaxin activation of its receptor RXFP1. The relaxin receptor RXFP1 can form heterodimers with the angiotensin II type 2 (AT2R), contribute to tissue-protective effects of relaxin. Relaxin antagonizes TGFβ signaling through activation of the protein kinase ERK1, with downstream activation of endothelial nitric oxide synthase (NOS1) resulting in increased nitric oxide (NO) production. This in turn activates soluble guanylyl cyclase (GC) and cGMP production. This pathway inhibits phosphorylation of Smad2/3, resulting in decreased TGFβ signaling. The RXFP1 signaling pathway also involves coupling to Gαs to promote activation of adenylyl cyclase (AC), cAMP production and activation of protein kinase A (PKA). Activated PKA phosphorylates and activates the transcription factor cAMP response element binding protein (CREB) to induce expression of the coactivator protein PPARγ coactivator 1α (PGC1α), which serves to increase PPARγ activity.

There is evidence of interplay between relaxin and other signaling pathways (**Figure 4**). It was recently shown that the relaxin receptor RXFP1 can form heterodimers with the angiotensin II type 2 (AT2R), and that renal-protective effects of relaxin required the presence and activity of AT2R (Chow et al., 2014). One major mechanism for relaxin’s antifibrotic effects is by antagonism of TGFβ signaling (Samuel et al., 2016a,b). Relaxin binding to RXFP1 results in activation of the protein kinase ERK1, with downstream activation of endothelial nitric oxide synthase (eNOS, or NOS1) and increased nitric oxide production. This in turn activates soluble guanylyl cyclase and cGMP production. This pathway has been shown to inhibit the phosphorylation of Smad2/3, resulting in decreased TGFβ signaling (Chow et al., 2012). An additional RXFP1 signaling pathway results from coupling to Gαs to promote activation of adenylyl cyclase, cAMP production and activation of protein kinase A (PKA). It was recently shown that this pathway, through phosphorylation of the transcription factor cAMP response element binding protein (CREB), promotes expression of the coactivator protein PPARγ coactivator 1α (PGC1α), which serves to increase PPARγ activity, providing potential cross-talk between these two antifibrotic pathways (Singh et al., 2015; Singh and Bennett, 2010). Relaxin activation of PPARγ activity has also been detected in brain arterioles (Chan and Cipolla, 2011; Chan et al., 2013). In support of this concept, a recent study showed that relaxin enhanced the response of other airway dilators, including the PPARγ agonist rosiglitazone (Lam et al., 2016).

## Summary and Perspective

The current options for the treatment of fibrotic diseases are extremely limited, and to date no effective drug has emerged that successfully targets established fibrosis. The four avenues of potential treatments discussed here show considerable progress, but to date have not translated to clinical treatment. Useful antifibrotic therapies must be effective against not only against reducing the excess collagen accumulation, but also accommodate collagen degradation. Ideally, the agents should have oral bioavailability. This presents challenges to peptide-based treatments, such as BMP7 or relaxin, and novel small molecule receptor agonists may hold the key to future therapeutics in these areas. In addition, and perhaps most importantly, off-target effects should be minimized, which might be overcome with nanoformulated preparations of the drugs. The novel agents provided in this review show promise as potential future treatments for fibroses, but more work is needed to determine if they will be ultimately translated to human disease.

## Author Contributions

All authors listed, have made substantial, direct and intellectual contribution to the work, and approved it for publication.

## Conflict of Interest Statement

The authors declare that the research was conducted in the absence of any commercial or financial relationships that could be construed as a potential conflict of interest.
